# New trends in regional anesthesia for shoulder surgery: Avoiding devastating complications

**DOI:** 10.4103/0973-6042.68410

**Published:** 2010

**Authors:** André P. Boezaart, Patrick Tighe

**Affiliations:** 1 Department of Anesthesiology, University of Florida College of Medicine, Gainesville, FL, USA; 2 Department of Orthopaedic Surgery & Rehabilitation, University of Florida College of Medicine, Gainesville, FL, USA

**Keywords:** Acute pain management, complications, continuous peripheral nerve block, permanent nerve damage, regional anesthesia, shoulder surgery, upper limb surgery

## Abstract

Surgeons and patients are often reluctant to support regional anesthesia (RA) for shoulder and other orthopedic surgeries. This is because of the sometimes true but usually incorrectly perceived “slowing down” of operating room turnover time and the perceived potential for added morbidity. Recently, severe devastating and permanent nerve injury complications have surfaced, and this article attempts to clarify the modern place of RA for shoulder surgery and the prevention of these complications. A philosophical approach to anesthesiology and regional anesthesiology is offered, while a fresh appreciation for the well-described and often forgotten microanatomy of the brachial plexus is revisited to explain and avoid some of the devastating complications of RA for shoulder surgery.

## INTRODUCTION

Every discipline in medicine has a basic fundamental truth that it ascribes to. For example, for critical care medicine (CCM), this truth is barrier integrity. In a healthy person, all anatomical and physiological barriers are intact and functional. These barriers are generally energy-dependent and keep critical substances separated. Upon death, all these barriers fail: fluid moves into the alveoli, bacteria move into the blood stream, and sodium and potassium equilibrate across cell membranes.

Anesthesia and regional anesthesia (RA) maintain their own peculiar truth. Understanding this truth may promote better understanding of what anesthesiologists instinctively or knowingly strive to accomplish. At the very least, such understanding may help the perioperative team to avoid some common anesthetic pitfalls.

## THE FUNDAMENTAL TRUTH OF ANESTHESIOLOGY AND REGIONAL ANESTHESIA

If CCM is about managing bariers, then anesthesia is about managing reflexes. These can be physiological reflexes; if one cuts the skin the patient reacts, if one places a tube in the trachea, the patient reacts, and so forth until surgery would be an exercise in frustration. During surgery and trauma, most of the reflexes are initiated by nociception, or the noxious stimuli resulting from surgery or trauma. Others are initiated by hypovolemia and other stimuli, but this review will focus on nociception and management of the reflexive response.

The physiological reactions to pain are vast and well known, and vary from attempting to get up off the table and run away, to an increased heart rate and blood pressure. These reflexes can be managed at the end organ, for example by giving beta-blocking agents to counteract the hypertension and tachycardia response to noxious stimuli. The use of muscle relaxants to prevent the spinal cord-mediated reflexive movement to nociception is another example.

A second place where this reflex to noxious stimuli can be interrupted is in the central nervous system. Using potent opioids such as fentanyl, alfentanil, remifentanil, and morphine to occupy receptors throughout the brain and spinal cord usually minimizes the reaction to such stimuli – a popular technique, perhaps, but not without cost in the form of unwanted and unpleasant side effects. Opioids bring with their administration the well-known side effects of tolerance, nausea, constipation, and respiratory depression, along with the lesser-known effects of possible immune suppression[1] and postoperative hyperalgesia.[2,3]

Perhaps the optimal place to interrupt these noxious impulses resides within the site of origin. Avoiding the painful stimulus in the first place can do this, but this is unrealistic in a surgical setting. Next best, then, is “hiding” the surgical stimulus from the ever-vigilant spinal cord. Physicians can block the nociception receptors from transmitting a message of pain to the brain by injecting local anesthetic agents into the surgical field, or by blocking the nerves that relay the message of pain to the spinal cord and brain. That is the prime use of RA in modern practice.

In modern anesthesiology, it is no longer a situation of general anesthesia (GA) versus regional anesthesia (RA). The out-dated notion that RA is safer than GA does not hold true anymore. Although literally thousands of research projects attempted to demonstrate that RA is safer than GA, these efforts were frustrated and consistently met with failure because the premise is simply not true. For example, in 1954 the mortality associated with GA was 3.7 per 1,000.[4] That means that for every 1,000 patients induced with GA in 1954, 3.7 would die as a direct result of the anesthesia. In 1982 that figure improved to a mere 1 per 10,000 anesthetics,[5] and it did at that time make sense to offer some alternative to GA. That was probably the reason for the sharp increase in the use of RA over the last half of the previous century. Now, in 2010, we can relax, GA is safe, and has an associated mortality rate of approximately 1:300,000.[6] We can now focus our energy on the appropriate management of pre-, intra-, and postoperative pain. RA is perfectly suited for this, because it is very effective, safe (if the time-tested rules are obeyed), can be administered preoperatively, used for intraoperative analgesia and continued through the postoperative period.

Modern anesthesiology, therefore, is all about managing reflexes associated with surgery, and to disrupt the pathway of the noxious stimulus to the brain makes perfect sense. This is the modern use of RA. In the postoperative period, RA provides excellent analgesia, while minimizing opioid-related side effects by reducing and even eliminating the need for opioids. RA has proven its value as the ultimate weapon in the armamentarium of multimodal approaches to postoperative pain.

## SINGLE-INJECTION VERSUS CONTINUOUS NERVE BLOCKS

Single-injection nerve blocks are well suited as the sole anesthetic for peripheral surgery such as to the hands, feet, and eyes under monitored anesthetic care (MAC) conditions and various degrees of sedation – from conscious to unconscious sedation. However, single injection nerve blocks have a short duration of action and are only of value for the intraoperative and direct postoperative period. In a very painful surgical procedure, such as rotator cuff repair, single-injection nerve block may be valuable in managing the intraoperative pain. Such nerve blocks even help in getting the patient out of the recovery room expeditiously.[7] Unfortunately, the patient receiving a single-injection nerve block for rotator-cuff repair is the same unfortunate soul waking at 0200 with severe, uncontrolled, and unmanageable pain in the dark and loneliness of the night. Most surgeons, and especially patients, would agree that in this situation it would probably have been better to have no block and to manage the pain with conventional analgesics before discharging the patient from the hospital or surgical center.

If, therefore, the patient is expected to suffer severe postoperative pain, it is the modern trend to place a continuous nerve block preoperatively. The anesthesiologist injects a bolus through the perineural catheter sufficient for an intraoperative surgical block, and then infuses a low concentration and volume of local anesthetic through the perineural catheter for a few days following the surgery.[8] However, ambulatory continuous nerve block requires continuous and costly involvement of healthcare professionals, and not all anesthesiologists are willing to spend such energy to add this liability without suitable remuneration. The question of who takes responsibility for the cost associated with ambulatory continuous nerve blocks has been unanswered, and some argue that the benefits are not worth the cost and effort. Although such systems are feasible, effective, and met with a high level of patient and surgeon satisfaction,[9–11] ambulatory continuous peripheral nerve blocks have unfortunately not yet reached their rightful place, except in the practices of a small number of enthusiastic anesthesiologists.

There is very little doubt that continuous nerve block is advantageous for in-patient care of patients, especially elderly patients, who require total joint replacement. Indeed, physicians seem to agree; 89% of French anesthesiologists would request a regional anesthetic for their own surgery, if the procedure were amenable to such a block.[12] Continuous nerve block has no place for relatively painless surgery such as carpal tunnel release and acromioplasty.[13] Here, the patient is burdened with the risks of a perineural catheter, whereas enjoying no benefit over single-injection nerve blocks or simple field block, which may be advantageous for these surgeries.

Is it possible to enjoy the prolonged analgesia of a perineural catheter, but absorb only the risk of a single-injection nerve block? Some workers have promoted the use of additives such as buprenorphine to the local anesthetic agent to extend the duration of action,[14] but this has not universally been met with enthusiasm. It is, generally, felt that if a long-acting block is needed a catheter should be placed for continuous nerve block, which is regarded by most as safer (or at the very least as safe) as single-injection blocks with fewer unwanted side effects or complications.

Why this apparent contradiction? With increasing concentrations of local anesthetics, nerves are generally blocked in the following order: pain fibers, then general sensory, and then motor.[15] The ideal local anesthetic would provide intense motor (also referred to as “surgical”) blockade during the intraoperative period. Shortly thereafter, the block’s intensity would decrease to cover only pain fibers, thus permitting full participation in rehabilitation while maintaining a pain- and opioid-free recovery. This pain-fiber coverage would last for 3 to 7 days before resolving completely, and the physician would have a mechanism for concluding the nerve block prematurely if clinically indicated.

Current mechanisms used to prolong single-injection nerve blocks do so by extending the entire “pharmacokinetic” profile of the nerve block. That is, each stage of motor, sensory, and analgesia block is prolonged. Prolonged motor, and even sensory, blocks run several risks, including suboptimal participation in rehabilitation, as well as neurapraxic injuries stemming from limb malpositioning unnoticed by patients in the days following surgery. In contrast, the properly managed perineural catheter permits intense surgical blockade during the intraoperative period, and then titratable analgesic-level nerve blockade for days thereafter, with the option of discontinuing the nerve block effect at any point. This has not been fully clarified by research, but at the very least, it can be accepted that continuous nerve block is as safe and free of side effects as single-injection nerve block.

Drug companies have also worked energetically for at least the past 15 years to develop a long-acting local anesthetic agent, but this cannot expect to be successful for anything but local wound infiltration, because similar to extended release epidural morphine, it is not only the wanted effects of analgesia that is long-lasting, the unwanted side effects, for example, phrenic nerve paralysis, will also be long-lasting. For those reason catheters for long-term nerve block are, for the time-being at least, here to stay.

## SEVERE NEUROLOGICAL COMPLICATIONS

One unpublished case of a young professional that permanently lost the function of the superior trunk of the brachial plexus (personal communication), and a number of unpublished cases of quadriplegia and at least two published reports of spinal cord injury[16] brought this issue acutely to the forefront.

### Radiating pain upon removal of a continuous nerve block catheter

The patient who lost the function of his superior trunk received an ambulatory continuous interscalene block for a rotator cuff repair. Upon removing the catheter at home the day after surgery, he experienced severe pain that radiated down his arm, which he described as a “lightning bolt going down his arm.” The anesthesiologists reassured him and he continued to remove the catheter, which ended with total and permanent loss of the fifth and sixth roots of the brachial plexus.

The catheter was advanced 15 cm beyond the tip of the needle, and was clearly curled around the superior trunk of the brachial plexus. Upon removal of the catheter, the nerve roots were avulsed, which caused the “lightning bolt” sensation experienced by the patient followed by the permanent nerve injury and loss of function.

We therefore strongly propose the following protocol for catheter removal:

- We do not condone the “bolus and pull” practice of some practitioners.
- Removal of a catheter can only take place when the sensation and motor function have fully returned to the limb.
- If there is no pain with removal of the catheter, it can safely be removed.
- If there is radiating pain, fluoroscopy with contrast of the plexus should be undertaken. Ultrasound examination of the brachial plexus could also be done, and, under both scenarios if light tugging on the catheter causes the brachial plexus to move with the catheter, surgical removal of the catheter is mandatory. If the brachial plexus does not move with the tugging on the catheter, the catheter can be removed carefully.

Surgical removal should be an extremely rare occurrence, but if indicated one should not hesitate to do it. With the modern recommendation of not advancing catheters further than 3–5 cm beyond the needle tip, it can be expected that this phenomenon will disappear.

### Spinal cord injury

Voermans *et al*. highlighted this problem.[16] Unfortunately, it is the humble opinion of the current authors that this problem could be expected to increase as the use of ultrasound increases. It is a fact that the brachial plexus is best visualized when the ultrasound probe is placed perpendicular to the nerves with the scalene muscles and nerves in the short axis of the ultrasound beam.[17] It is also a fact that the in-plane approach to these nerves is the easiest way to perform a brachial plexus block.[18,19] This leads to the so-called “posterior approach to the brachial plexus,” which is similar to the Pippa approach popular in Europe.[20] This, unfortunately, often leads to the block being done more medially and on the root level of the brachial plexus. This root-level approach provides an excellent analgesic block, but it is not readily appreciated that at this level the roots are surrounded by dura and penetration of the dura leads to a subdural injection, which can also be intramedullary, because the nerve tissue here is nothing but the axons originating in the spinal cord, which are accompanied by perineural spaces in which the local anesthetic agent spreads centrally. Both these scenarios can of course lead to devastating complications and preventing these can only be done if the microanatomy of the brachial plexus is fully understood. A review of this microanatomy follows.

## MICROANATOMY

Our understanding of the microanatomy of the peripheral nervous system is not new. Key and Retzius[21] in 1876 used Richardson’s stain, whereas Horster and Whitman[22] in 1931 used trypan blue to study the spread of intraneurally injected solutions experimentally. In more “recent” times, French *et al*. in 1948[23] studied intrafascicular injection with radiopaque contrast medium in dogs. Since this early work, even after the introduction of electron microscopy,[24] no new insights have been introduced to refute these concepts or add significant new knowledge.

### Peripheral nerve microanatomy

**Figure 1:**
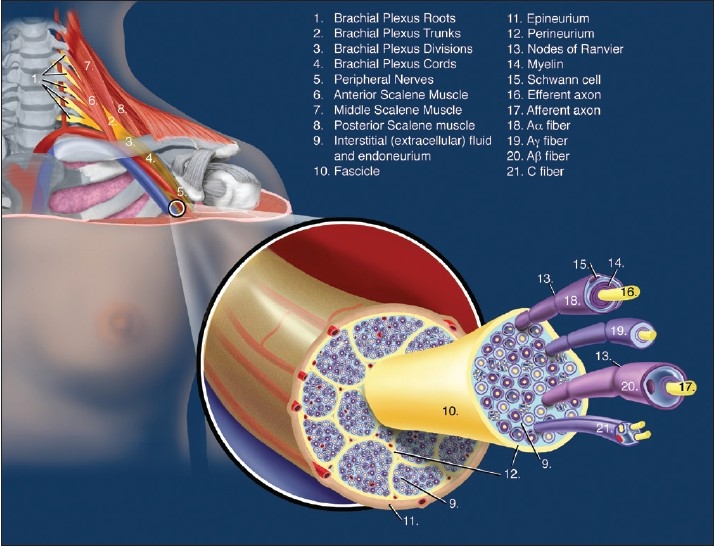
Microanatomy of a peripheral nerve

The embryological formation of the branches or peripheral nerves occurs later than roots and trunks, but for ease of understanding, the peripheral nerves will be considered first. Peripheral nerves are composed of numerous fasciculi; each surrounded by a dense perineurium and held together by a looser epineurium [Figure 1].

The *epineurium* consists of a condensation of areolar connective tissue that surrounds the perineurial ensheathment of the fascicles of uni- and multifascicular nerves.[25] The attachment of the epineurium to surrounding connective tissue is loose, so that the nerve is relatively mobile except where tethered by entering blood vessels or by branches.[25] Greater amounts of connective tissue are normally present where nerves cross the joints. In general, the more fascicles that are present, the greater the quantity of epineurium. Variable quantities of fat in the epineurium have a protective function in cushioning the fascicles against injury,[25] and the vasa nervorum enter the *epineurium*, where they communicate with a longitudinal anastomotic network of arterioles and venules.[25]

The *epineurium* also contains lymphatic vessels, which are not present within the fascicles. These lymphatic channels accompany the arteries of the peripheral nerves and pass into the regional lymph nodes.[25]

The essential structure of the *perineurium* is a lamellated arrangement of flattened cells separated by layers of collagenous connective tissue (similar to the plexus sheaths and, although not yet shown, one wonders if the epineurium is not a continuation of the plexus sheaths).[21] It provides an ensheathment for both the somatic and peripheral autonomic nerves and their ganglia. The cellular lamellae are composed of concentric sleeves of flattened polygonal cells, and these cells are equipped to function as a metabolically active diffusion barrier, although they do not have the morphologic features of a true epithelium.

The term *endoneurium* is sometimes erroneously used to denote the intrafascicular compartment of the nerve; it should only be used to refer to intrafascicular connective tissue, excluding the perineurial partitions that may subdivide fascicles.[25] Approximately 40–50% of the intrafascicular space is occupied by non-neural elements and about 20–30% of this is the endoneural fluid (CSF) and connective matrix (*endoneurium*).[25]

Longitudinal flow within the fascicle is inhibited minimally, whereas lateral extension is restricted by the relatively non-compliant perineurium.[25] As the nerve approaches the dural penetration, resistance to extension increases and a peripherally injected medium comes to lie in clefts in the perineurium. Final emergence into the subarachnoid space occurs first by way of the subdural space and subsequently by breakthrough across the arachnoid barrier into the subarachnoid space.

A fluid deliberately or accidentally injected into a fascicle of a peripheral nerve has direct access to the cerebrospinal fluid (CSF) and interstituim (medulla) of the spinal cord, and such spread depends directly on the volume and pressure applied.[26] The channels by which this progression occurs have been called perineurial spaces, and these have been previously demonstrated.[26] Injection into a spinal root, on the other hand, is easy, and this injectate, similarly has direct access to the CSF and spinal cord interstitium – the clinical consequences of which depends on the volume, rate, and pressure of the injectate and the path taken via the perineurial spaces of the axons. The injectate will generally follow the route of least resistance *via* these perineural spaces.[26]

Experimental work of Selander and Sjöstrand on intraneural injections into rabbit sacral nerves demonstrated that, during injection deep to the *epineurium* but outside the perineurium, an irregular bleb formed around the injection site.[25] The tracer that they injected spread for a short distance within an easily expanding epineurium, which often ruptured. When 50–100 µL were injected at 100 µL/min, the injection pressure rose within a few seconds to 30–60 mmHg and thereafter quickly decreased to a steady 10–30 mmHg. As soon as the injection stopped, the pressure returned to zero. During *intrafascicular* injection deep to the perineurium, however, the tracer was seen to spread rapidly, proximally, and distally inside the fascicle. The longitudinal spread varied, but in all cases, it reached the sacral plexus. Distally, the tracer colored the tibial nerve, sometimes even reaching the foreleg. In another study,[23] the tracer reached the lumbar plexus *via* the injected fascicle, and then even sometimes tracked distally *via* an entirely different nerve originating from the plexus. This study also showed that high-pressure intrathecal injection of contrast medium spread down the fascicles of peripheral nerves.

Selander *et al*.[26] demonstrated that if the injection was made into a small fascicle, the injectate did not extend beyond the sacral plexus, but if the injection was made into a big fascicle, the injectate easily passed the sacral plexus and reached the spinal cord. During slow injection, the spread in the medulla was superficially under the pia mater. In some of the experimental animals, the spread was into the CSF, and the dura and arachnoid were also colored. In one animal, the blue stain extended to the cerebellum. In cross sections of the spinal cord, the fluorescent tracer used was mainly seen is the thin sub-pial space.[26] Accumulation of the tracer was noted in the dorsal root-medulla junction area, extending into the *substantia gelatinosa* of the anterior horns, and into the anterior median fissure. They recorded pressures of between 435 and 675 mmHg when injecting 50–100 µL with a rate of 100 µL/min into a fascicle. After cessation of the injection, the pressure remained above the estimated capillary perfusion pressure (50 mmHg) for at least 10 min.

### Plexus trunk microanatomy

**Figure 2:**
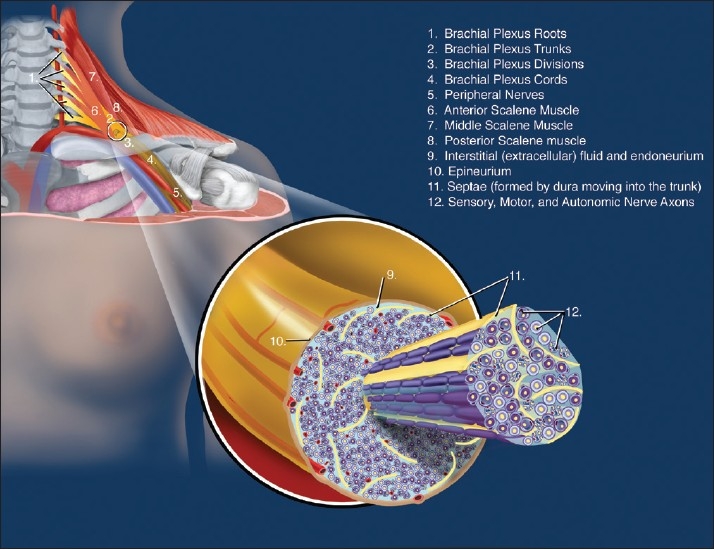
Microanatomy at the brachial plexus trunk level

The trunks of the plexuses are transitional areas [Figure 2].[23] The perineurium surrounding the fascicles split away and axons are separated by perineurial sheath interdigitations or septae. There seems to be inter-individual variation on the level at which the septae form, but functionally and practically from a RA perspective, the trunks should be regarded as transitional areas between clearly defined fasciculi with rigid perineuria at the branches to the root area where perineuria are not present and all the perineuria have joined to form the dura.[23]

### Plexus root microanatomy

**Figure 3:**
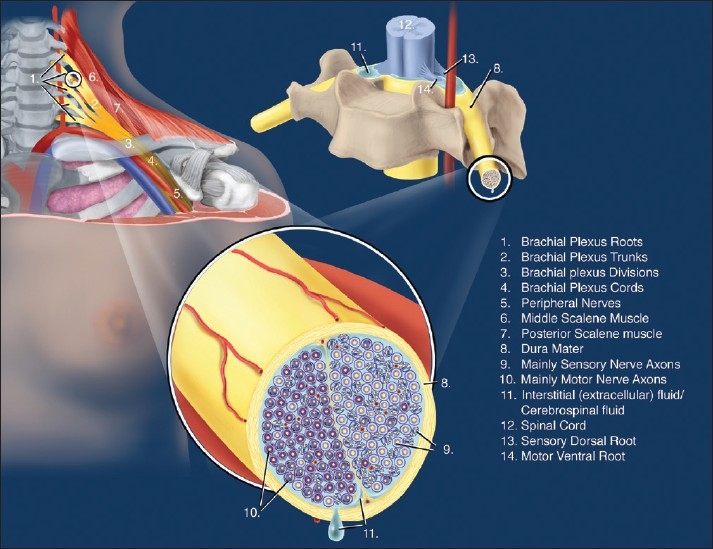
Microanatomy at the brachial plexus root level

After splitting away from the fascicles, at the level of the nerve roots, the perineurium thickens and fuses with the dura [Figure 3].[23,25] (Embryologically more correct, the peripheral nerve perineurium is a continuation of the dura mater.) The axons inside the roots are consequently not protected by the perineurium anymore and the extracellular or tissue fluid is the CSF. The connective tissue framework of the peripheral nervous system, therefore, arises entirely from the dura mater to a continuation of the perineurium around the fascicles of the branches. As the nerve progresses peripherally, it is more and more subdivided by perineural interdigitations until each fascicle of nerve axons eventually has its own perineurial sheath. The mesothelial cells of the arachnoid membrane become hyperplastic where they exit the nerves and form a cuff around the roots just after they penetrate the dura mater.[25] Beyond this cuff, no tissue can be seen that is recognized as arachnoid.

### Ultrasound

**Figure 4:**
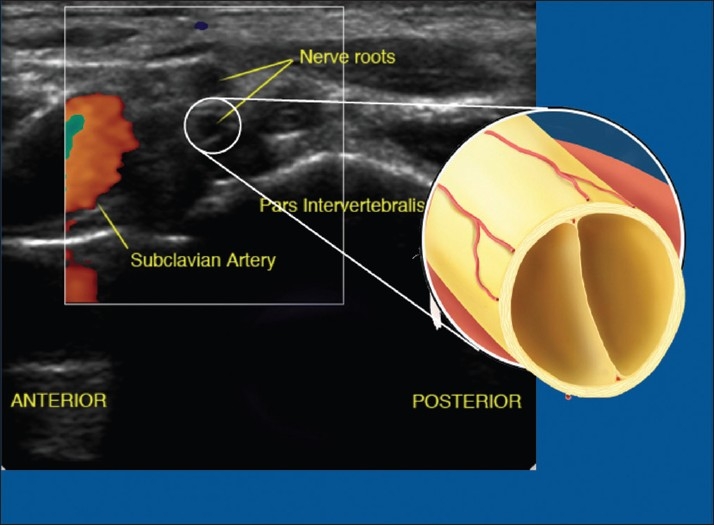
Ultrasound picture at the brachial plexus root level. Note root appears hypo-echoic

**Figure 5:**
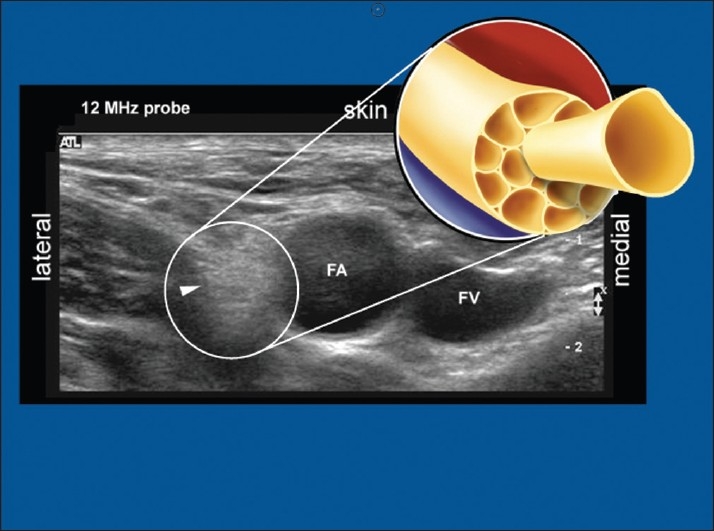
Ultrasound appearance of the femoral nerve – a peripheral nerve. Note the nerve appears hyper-echoic

With the recent introduction of *ultrasound to regional anesthesia*, it became clear that nerves could either be hyper- or hypo-echoic.[17] When studying the ultrasonographic appearance, it can be seen that the more proximal the nerve the more hypo-echoic (black appearance) [Figure 4] and the more distal, the more hyper-echoic the nerve (“honeycomb” appearance) [Figure 5]. With the insight of the nerve microanatomic morphology, this should be easy to understand in practical terms – even if not entirely correct in pure physics terms [Figures 4 and 5].

### Intraneural injection

Although intraneural but extra-fascicular injection at the branch level is probably without consequences,[27] injections at the root level (and perhaps trunk level of some individuals) should be regarded as epidural injections, because the injection is made directly outside the dura-extra-dural, peri-dural, or epidural.[28] All the time-tested safety practices for spinal epidural injections should therefore similarly apply for root level or paraneuraxial or paraspinal extra- or epidural injections. These should include the use of large bore relatively blunt Tuohy needles and the avoidance of sharp thin needles (for continuous and single-injection blocks)[28]; the use of similar test doses to test for intravascular or intrathecal injection, fractionation of the main dose, and perhaps even similar guidelines for anticoagulation, although this is open to debate and can be expected to be further contested.[29] All the catastrophic, potentially catastrophic, and tragic cases, ranging from total spinal block to quadriplegia, referred to in the papers by Antonakakis *et al*.[18] and Mariano *et al*.[19] can comfortably be explained by intraroot (sub-dural) injections with relatively thin and sharp needles that were not designed for use around the dura. All the presented cases were spinal root or trunk level blocks[30] performed with needles that one would not use for a spinal epidural block. All root level blocks (cervical, thoracic, lumbar, and sacral), and perhaps even trunk level blocks, such as interscalene blocks in certain individuals,[30] should probably be regarded and respected as *para-spinal or para-neuraxial epidural blocks* similar to *spinal epidural or neuraxial blocks* to afford it the appropriate level of respect that will avoid disastrous complications.

## CONCLUSION

Modern anesthesiology should be regarded as the science of managing reflexes. The reflexes following noxious surgical stimuli can and should be managed at its origin rather than at its end organ response. In this regard, RA plays a pivotal roll in managing the reaction to intraoperative painful stimuli by preventing it from reaching the spinal cord and brain. Postoperatively, RA is also of great value to treat pain following surgery and trauma and to minimize the use of opioids.

It is clear that institutions benefits greatly from single-injection nerve block for postoperative pain management, and it is also clear that, except for the practices of a few dedicated enthusiasts, ambulatory continuous nerve block is highly beneficial to patients, but the logistical and financial aspects of it is far from solved.

Severe and permanent nerve injuries have been forced to the forefront, but with careful catheter removal after full sensation has returned to the limb and with full understanding and respect for the microanatomy of the peripheral nervous system, these complications can and should be nullified. This article attempted to highlight the modern philosophical approach to anesthesiology for shoulder and other surgeries, and attempted to offer a practical approach to catheter removal to minimize or nullify nerve injury. Finally, it attempted to revisit the microanatomy of the brachial plexus, to caution practitioners of the microanatomical reasons for devastating complications.
